# Routine family history inquiry among family physicians: associations with perceived clinical usefulness and barriers: a cross-sectional study

**DOI:** 10.3389/fmed.2026.1794144

**Published:** 2026-04-14

**Authors:** Melike Karabulut Özer, Dilara Canbay Özdemir

**Affiliations:** Department of Family Medicine, Ordu University Faculty of Medicine, Ordu, Türkiye

**Keywords:** family history, family physicians, primary care, risk assessment, clinical usefulness, perceived barriers

## Abstract

**Objective:**

Family history is a core component of individualized risk assessment in primary care, particularly for cardiovascular disease, hypercholesterolemia, and cancer; however, it is not always routinely obtained in everyday practice. This study aimed to describe family physicians’ self-reported family history inquiry behavior and to examine its associations with perceived clinical usefulness, perceived barriers, and inquiry into selected heritable conditions.

**Materials and methods:**

This cross-sectional, self-administered online survey included 257 family physicians practicing in primary care in Türkiye. The questionnaire assessed the frequency of family history inquiry, perceived clinical usefulness, perceived barriers, and the extent of inquiry into selected heritable conditions. Two author-developed Likert-type scales were used as exploratory composite measures. Non-parametric tests, chi-square analyses, Spearman’s correlation analysis, and univariable binary logistic regression analyses were performed.

**Results:**

Routine family history inquiry was self-reported as being performed frequently by 55.3% of participants and occasionally by 41.2%. Higher perceived clinical usefulness scores were associated with more frequent self-reported inquiry. Perceived clinical usefulness and perceived barriers showed a moderate positive correlation (Spearman’s rho = 0.341, *p* < 0.001). In the logistic regression analysis, perceived clinical usefulness remained associated with frequent rather than occasional self-reported family history inquiry (OR: 1.36, 95% CI: 1.01–1.83, *p* = 0.045).

**Conclusion:**

In this cross-sectional survey of family physicians, more frequent self-reported routine family history inquiry was associated with a higher perceived clinical usefulness of family history information. These findings indicate self-reported behavior and perceived utility and should not be interpreted as evidence of causal effects or measured effects on clinical decision-making outcomes.

## 1 Introduction

Family history is a composite measure of shared genetic susceptibility, environmental exposures, and behavioral patterns, and it remains a cornerstone of individualized risk assessment in clinical practice. Its relevance is particularly well-established for chronic conditions, such as premature cardiovascular disease, hypercholesterolemia, and several common cancers, in which familial aggregation may substantially modify individual risk profiles and screening needs (1–4).

In cardiovascular medicine, international guidelines emphasize family history as an essential component of global risk stratification, particularly for identifying individuals with elevated lifetime risk who may not yet meet traditional risk thresholds. Similar principles apply to hereditary cancer syndromes and familial hypercholesterolemia, where structured family history assessment can guide earlier surveillance, targeted referral, and personalized preventive strategies (5–11).

In addition to general risk stratification, family history may also influence both organized and opportunistic screening decisions in primary care, helping physicians identify individuals who could benefit from earlier, more tailored, or more intensive screening pathways (8, 12, 13).

Primary care physicians, particularly family physicians, are uniquely positioned to collect, interpret, and act upon family history information because of their longitudinal patient relationships and comprehensive care perspective. However, previous studies have demonstrated substantial variability in how frequently and systematically family history is obtained in routine primary care, and reported barriers include time constraints, uncertainty regarding clinical utility, and lack of standardized tools (14–18).

Although the conceptual importance of family history is well documented, evidence regarding physicians’ routine inquiry behaviors and the factors influencing these behaviors remains limited, particularly in relation to perceived clinical usefulness and perceived barriers. In addition, the extent to which inquiry into selected heritable conditions is associated with more consistent family history taking has not been sufficiently clarified (13, 19).

The present study aimed to describe family physicians’ self-reported routine family history inquiry habits and to explore the associations between routine inquiry behavior and perceived clinical usefulness, perceived barriers, and inquiry into selected heritable conditions. By exploring these relationships, this study aimed to contribute to a more nuanced understanding of the factors shaping family history use in primary care practice.

## 2 Materials and methods

### 2.1 Study design and participants

This study was designed as a cross-sectional, questionnaire-based survey conducted to describe family physicians’ self-reported family history inquiry practices, their perceived clinical usefulness of family history information in practice, and perceived barriers to its routine use.

The questionnaire was disseminated between 1 October 2025 and 30 November 2025 through an open online survey link targeting physicians actively providing primary care services in Türkiye. Since the survey was shared as an open and forwardable link, the exact number of physicians who received or viewed the invitation could not be determined; therefore, a conventional response rate could not be calculated.

The survey was self-administered. Before accessing the questionnaire items, participants were presented with an electronic informed consent statement and could proceed only after indicating that they had read and accepted the consent information. A total of 257 responses were available for analysis. Fully completed questionnaires were included in the main analyses, and partially completed responses were considered on a variable-specific basis when appropriate.

A brief STROBE-style flow summary is as follows: open online link disseminated → responses received (*n* = 257) → records with analyzable data included in descriptive analyses (*n* = 257) → participants reporting at least occasional family history inquiry included in the frequent–occasional regression analyses (*n* = 248).

### 2.2 Questionnaire content

The questionnaire assessed sociodemographic and professional characteristics, routine frequency of family history inquiry, perceived clinical usefulness, perceived barriers, and the extent to which respondents inquired about selected heritable conditions in clinical practice.

The full questionnaire covered a broader range of disease groups (Supplementary File 1). However, the main inferential analyses focused *a priori* on early-onset cardiovascular disease, hypercholesterolemia, and cancer because these domains were considered especially clinically actionable and screening relevant in primary care.

Professional title/specialist status was recorded as a descriptive participant characteristic.

### 2.3 Perceived Clinical Usefulness Scale

A Likert-type Perceived Clinical Usefulness Scale was used to assess physicians’ perceptions of how useful family history information is in clinical practice. The scale comprised four items: risk assessment, diagnostic testing and screening decisions, treatment initiation/intensity, and referral/genetic counseling decisions.

Each item was scored from 1 (strongly disagree) to 5 (strongly agree). The Perceived Clinical Usefulness Scale score was calculated as the arithmetic mean of the four items and was used as a continuous variable in the analyses.

This scale was developed by the research team on the basis of the study aims and relevant literature and was used as an exploratory composite measure rather than as a previously validated external instrument (20).

### 2.4 Perceived Barriers Scale

A second Likert-type scale was used to evaluate barriers encountered in the routine clinical use of family history. The Perceived Barriers Scale included time constraints, the reliability of patient-provided information, the lack of an appropriate documentation field/template, access to current guideline information, and privacy/ethical concerns.

Each item was scored from 1 (not at all) to 5 (very much). The scale score was calculated as the arithmetic mean of the five items and was used as the perceived barrier score in the analyses.

Similar to the Perceived Clinical Usefulness Scale, this measure was developed by the research team for the present study and should be interpreted as an exploratory, author-developed composite measure rather than a fully validated psychometric instrument.

### 2.5 Statistical analysis

Statistical analyses were performed using IBM SPSS Statistics version 27. Categorical variables were summarized as numbers and percentages, and continuous variables were summarized as the mean ± standard deviation or the median [Q1–Q3] according to distributional characteristics. Internal consistency was evaluated using Cronbach’s alpha, and item-total correlations were examined for the author-developed scales.

As the scale scores were not normally distributed, non-parametric methods were used. The Mann–Whitney U-test was used for two-group comparisons, chi-square tests were used for categorical associations, and Spearman’s correlation analysis was used to examine relationships between continuous or ordinal variables.

To clarify the analytical framework, a univariable binary logistic regression model was performed only among physicians who reported at least occasional family history inquiry, because data on the clinical usefulness score were not available for those who reported no inquiry due to questionnaire design. The binary outcome was the self-reported frequency of routine family history inquiry, coded as frequent inquiry = 1 and occasional inquiry = 0, and the clinical usefulness score was entered as a continuous independent variable. Odds ratios (ORs) with 95% confidence intervals (CIs) were reported. Statistical significance was set at a *p*-value of < 0.05.

### 2.6 Ethical considerations

The study was conducted in accordance with the Declaration of Helsinki and was approved by the Ordu University Clinical Research Ethics Committee (approval no. 2025/320).

Electronic informed consent was obtained from all participants before questionnaire completion. No directly identifiable personal data were collected.

## 3 Results

A total of 257 family physicians were included in the descriptive analyses. Participant characteristics are presented in Table 1. The median age was 35.0 years (30.0–40.0), and 132 (51.4%) participants were male. Professional titles were heterogeneous, with the largest groups being SAHU assistants (a government-contracted family medicine specialty training program specific to Türkiye) and family medicine residents.

**Table 1:** Participant characteristics and routine family history inquiry habits (*n* = 257).

Characteristic	Category/summary	*n* (%)
Age (years)	Median	35.0 (30–40)
Sex	Female	125 (48.6)
Sex	Male	132 (51.4)
Professional title	SAHU resident*	85 (33.1)
Professional title	Family medicine resident	77 (30.0)
Professional title	Family medicine specialist	51 (19.8)
Professional title	General practitioner	44 (17.1)
Years working as a family physician	0–5 years	127 (49.4)
Years working as a family physician	6–10 years	58 (22.6)
Years working as a family physician	11–20 years	62 (24.1)
Years working as a family physician	≥21 years	10 (3.9)
Routine family history inquiry	No	9 (3.5)
Routine family history inquiry	Yes, sometimes	106 (41.2)
Routine family history inquiry	Yes, frequently	142 (55.3)

*SAHU: a government-contracted family medicine specialty training program specific to Türkiye.

More than half of the participants reported that they frequently inquired about family history in routine practice, whereas 41.2% reported doing so occasionally, and only a small minority reported never doing so.

In three-category analyses, physicians who reported asking about familial early-onset cardiovascular disease, hypercholesterolemia, and cancer also reported more frequent routine family history inquiry overall (Table 2). When the comparison was restricted to physicians who already reported at least occasional inquiry, asking about familial hypercholesterolemia remained associated with frequent rather than occasional inquiry, whereas the difference for familial cancer was attenuated.

**Table 2:** Inquiries about selected familial conditions according to the routine family history inquiry category.

Selected condition asked about	No	Yes, sometimes	Yes, frequently	Chi-square	*p*
Early-onset cardiovascular disease	0 (0.0)	101 (39.3)	132 (51.4)	90.93	<0.001
Hypercholesterolemia	0 (0.0)	72 (28.0)	115 (44.7)	30.14	<0.001
Cancer	0 (0.0)	87 (33.9)	127 (49.4)	48.78	<0.001

Values are presented as the *n* (%) of the total study sample (*N* = 257), not as column percentages. For each selected condition, the counts indicate the number of physicians who reported asking about that condition within each routine family history inquiry category. Participants reporting no routine family history inquiry contributed no affirmative responses to these selected-condition items in the analyzed dataset.

The four-item Perceived Clinical Usefulness Scale demonstrated high internal consistency (Cronbach’s alpha = 0.909), with item-total correlations ranging from 0.720 to 0.845. The five-item Perceived Barriers Scale showed acceptable internal consistency (Cronbach’s alpha = 0.770), with item-total correlations ranging from 0.433 to 0.595 (Table 3). Item-level descriptive results are provided in Supplementary Tables 1, 2.

**Table 3:** Descriptive statistics and reliability of the author-developed scales.

Scale	Mean ± SD	Median (Q1–Q3)	Minimum–Maximum	Cronbach’s alpha
Perceived Clinical Usefulness Scale score	4.07 ± 0.85	4.00 [3.50–5.00]	1.00–5.00	0.909
Perceived Barriers Scale score	3.29 ± 0.82	3.20 [2.80–3.80]	1.40–5.00	0.770

Both scales consisted of author-developed exploratory composite measures. Scale scores were calculated as the arithmetic mean of the item scores, with higher values indicating greater perceived clinical usefulness or greater perceived barriers, respectively.

The mean Perceived Clinical Usefulness Scale score was 4.07 ± 0.85, and the mean Perceived Barriers Scale score was 3.29 ± 0.82. Both scores deviated significantly from normality (Shapiro–Wilk test, *p* < 0.001 for both).

A moderate, positive, and statistically significant correlation was observed between perceived clinical usefulness and perceived barriers (Spearman’s rho = 0.341, *p* < 0.001), indicating that physicians who perceived greater clinical usefulness of family history also tended to report higher levels of perceived implementation barriers.

Among physicians who reported at least occasional family history inquiry, those who reported frequent inquiry had significantly higher Perceived Clinical Usefulness Scale scores than those who reported occasional inquiry (Mann–Whitney U-test, *p* = 0.020).

In the univariable binary logistic regression analysis (Table 4), restricted to physicians who reported at least occasional family history inquiry, a higher Perceived Clinical Usefulness Scale score was associated with higher odds of reporting frequent rather than occasional inquiry (OR: 1.36, 95% CI: 1.01–1.83, *p* = 0.045).

**Table 4:** A univariable binary logistic regression analysis predicting frequent versus occasional self-reported family history inquiry among physicians reporting at least occasional inquiry (*n* = 248).

Predictor	B (SE)	OR	95% CI for OR	*p*
Perceived Clinical Usefulness Scale score	0.306 (0.153)	1.36	1.01–1.83	0.045

Outcome variable: self-reported routine family history inquiry frequency, coded as frequent inquiry = 1 and occasional inquiry = 0. The predictor was the Perceived Clinical Usefulness Scale score, entered as a continuous variable. Participants reporting no routine family history inquiry were excluded because this score was unavailable for that group by questionnaire design. No multivariable model was fitted.

## 4 Discussion

In this cross-sectional survey of family physicians, more than half of the respondents reported frequently asking about family history, whereas a substantial proportion reported doing so only sometimes. These findings are consistent with prior reports suggesting that family history collection in primary care is often recognized as important but not always implemented systematically (14, 17).

One of the central findings of the present study was the association between more frequent self-reported family history inquiry and higher perceived clinical usefulness of family history information. Physicians who assigned greater practical usefulness to family history in relation to risk assessment, testing or screening, treatment decisions, and referral considerations also reported more routine inquiry. Given the cross-sectional design and the self-reported nature of the data, this finding should be interpreted as an association between perceived usefulness and reported behavior rather than as evidence of a causal relationship or of measured effects on clinical decision-making outcomes.

The findings related to specific disease domains were also informative. Inquiry about familial history of cardiovascular disease and hypercholesterolemia clustered with more routine family history inquiry overall, which is clinically plausible because these conditions are readily translated into cardiovascular prevention and risk communication workflows in primary care (5, 9, 13). Inquiry about family history of cancer also showed an overall association across inquiry categories, but this difference was less clear when the analysis was restricted to frequent versus occasional inquirers. This attenuation may reflect greater heterogeneity in how physicians operationalize cancer-related family history in routine practice, including uncertainty about referral thresholds and screening pathways (10, 11, 19).

The positive correlation between perceived clinical usefulness and perceived barriers is noteworthy. Physicians who were more aware of the clinical value of family history also tended to perceive more barriers. Rather than being contradictory, this pattern may indicate that clinicians who engage more seriously with family history are also more sensitive to the time, documentation, and workflow limitations that constrain implementation (15–18).

From a preventive care perspective, family history remains conceptually relevant for risk stratification and screening in primary care (8, 12, 13). In the present study, however, the data address physicians’ self-reported inquiry behavior and perceived usefulness rather than actual downstream screening practices, referrals, or patient outcomes. Accordingly, the findings should be interpreted as reflecting how physicians perceive and report the use of family history in routine practice.

The present findings may be useful for hypothesis generation regarding how inquiry about family history is perceived and reported in primary care settings. In particular, they suggest that perceived practical usefulness and implementation barriers coexist and may both be relevant when considering future research on structured family history approaches in routine care.

### 4.1 Limitations

This study has several limitations. First, its cross-sectional design does not permit causal inference. Second, all measures were based on physician self-report and are therefore subject to recall, reporting, and social desirability biases. Third, because the survey was disseminated via an open, forwardable online link, the exact number of physicians who received or viewed the invitation was unknown; as a result, a conventional response rate could not be calculated, and selection effects related to open recruitment and volunteer participation may limit representativeness. Fourth, the Perceived Clinical Usefulness and Perceived Barriers scales were author-developed exploratory composite measures rather than fully validated external instruments, which may affect measurement precision, comparability, and generalizability. Finally, since the study assessed self-reported inquiry behavior and perceived usefulness rather than observed practice patterns, downstream screening actions, referrals, or patient outcomes, the findings should be interpreted cautiously and should not be taken as evidence of actual clinical effectiveness.

## 5 Conclusion

In this cross-sectional survey, higher perceived clinical usefulness of family history information was associated with more frequent self-reported routine family history inquiry among family physicians. These findings reflect self-reported behavior and perceived utility rather than measured effects on clinical decision-making or patient outcomes. Supporting routine family history use in primary care may require both practical workflow facilitation and educational strategies that address how family history information can inform risk assessment, screening considerations, treatment discussions, and referral processes.

## Data Availability

The raw data supporting the conclusions of this article will be made available by the authors, without undue reservation.
