# Supplementary material for: Routine family history inquiry among family physicians: associations with perceived clinical usefulness and barriers: a cross-sectional study
Source: Front Med (Lausanne). 2026 Apr 14;13:1794144. doi: 10.3389/fmed.2026.1794144 (PMC13120918; doi:10.3389/fmed.2026.1794144)
Supplement: Supplementary file 1 [file Data_Sheet_1.DOCX]

**Supplementary Table 1. Item-level descriptive summaries for the Perceived Clinical Usefulness** **Scale.**

| **Item** | **Mean +/- SD** | **Median [Q1-Q3]** | **Item-total correlation** | **Observed distribution (score: n)** |
| --- | --- | --- | --- | --- |
| Family history meaningfully affects my risk assessment. | 4.18 +/- 0.91 | 4.00 [4.00-5.00] | 0.835 | 1:1; 2:11; 3:45; 4:76; 5:115 |
| Family history affects my testing/screening decisions. | 4.15 +/- 0.92 | 4.00 [4.00-5.00] | 0.845 | 1:3; 2:11; 3:38; 4:89; 5:107 |
| Family history affects treatment initiation/intensity. | 3.78 +/- 1.07 | 4.00 [3.00-5.00] | 0.720 | 1:5; 2:25; 3:69; 4:70; 5:79 |
| Family history affects referral/genetic counseling decisions. | 4.17 +/- 0.93 | 4.00 [4.00-5.00] | 0.797 | 1:3; 2:9; 3:45; 4:78; 5:113 |

Overall Perceived Clinical Usefulness Scale reliability: Cronbach's alpha = 0.909.
