# Supplementary material for: Routine family history inquiry among family physicians: associations with perceived clinical usefulness and barriers: a cross-sectional study
Source: Front Med (Lausanne). 2026 Apr 14;13:1794144. doi: 10.3389/fmed.2026.1794144 (PMC13120918; doi:10.3389/fmed.2026.1794144)
Supplement: Supplementary file 2 [file Data_Sheet_2.DOCX]

**Supplementary Table 2. Item-level descriptive summaries for the Perceived Barriers Scale.**

| **Item** | **Mean +/- SD** | **Median [Q1-Q3]** | **Item-total correlation** | **Observed distribution (score: n)** |
| --- | --- | --- | --- | --- |
| Time constraint | 4.05 +/- 1.07 | 4.00 [3.00-5.00] | 0.433 | 1:4; 2:21; 3:53; 4:59; 5:120 |
| Reliability of patient-provided information | 3.49 +/- 1.02 | 4.00 [3.00-4.00] | 0.535 | 1:9; 2:30; 3:89; 4:84; 5:45 |
| Lack of an appropriate documentation field/template | 3.32 +/- 1.13 | 3.00 [3.00-4.00] | 0.569 | 1:19; 2:39; 3:79; 4:81; 5:39 |
| Access to up-to-date guideline information | 2.97 +/- 1.19 | 3.00 [2.00-4.00] | 0.595 | 1:30; 2:65; 3:73; 4:60; 5:29 |
| Privacy/ethical concerns | 2.64 +/- 1.24 | 3.00 [2.00-4.00] | 0.574 | 1:56; 2:68; 3:68; 4:42; 5:23 |

Overall Perceived Barriers Scale reliability: Cronbach's alpha = 0.770.
