# Supplementary material for: Routine family history inquiry among family physicians: associations with perceived clinical usefulness and barriers: a cross-sectional study
Source: Front Med (Lausanne). 2026 Apr 14;13:1794144. doi: 10.3389/fmed.2026.1794144 (PMC13120918; doi:10.3389/fmed.2026.1794144)
Supplement: Supplementary file 3 [file Supplementary_file_1.DOCX]

Supplementary File 1. Full questionnaire used in the study, translated into English from the original Turkish form. The wording below is presented to facilitate review and interpretation of the author-developed scales.

# Consent and demographics

| **Item** | **Question** | **Response options** |
| --- | --- | --- |
| 1 | I have read the information above, understood it, and agree to participate. | Response option: check box |
| 2 | Age | Open numeric response |
| 3 | Sex | Female / Male |
| 4 | Professional title | Family medicine specialist / General practitioner family physician / Family medicine resident / SAHU resident |
| 5 | Province of practice | Open text response |
| 6 | How many years have you been working as a family physician? | 0-5 years / 6-10 years / 11-20 years / >=21 years |
| 7 | Daily average number of patient visits | <40 / 40-59 / 60-100 / >=100 |

# General views on family history

| **Item** | **Question** | **Response options** |
| --- | --- | --- |
| 8 | How important do you consider asking about familial disease history in patients' health records? | Not important at all / Slightly important / Moderately important / Very important / Extremely important |
| 9 | Do you think family history should be given more importance than it currently receives? | Yes / No |
| 10 | Is there a disease with familial transmission in your own family? | Yes / No |
| 11 | If yes, which condition(s)? | Multiple choice |
| 12 | Do you personally have a disease with familial transmission? | Yes / No |
| 13 | If yes, which condition(s)? | Multiple choice |

# Routine inquiry practices

| **Item** | **Question** | **Response options** |
| --- | --- | --- |
| 14 | Do you routinely ask your patients about family history? | Yes, frequently / Yes, sometimes / No |
| 15 | In which patients do you ask about family history more often? | Multiple choice |
| 16 | Approximately in what proportion of your patients do you ask about family history? | <25% / 25-50% / 51-75% / 76-100% |
| 17 | How do you collect familial disease information? | Multiple choice |
| 18 | How much time do you spend on family history on average? | <2 min / 2-5 min / 6-10 min / >10 min |
| 19 | Which degrees of relatives do you ask about? | Multiple choice |
| 20 | Which disease groups do you ask about most often? | Multiple choice |
| 21 | How often do you update family history? | Rarely / Annually / At every important visit / When symptoms/diagnosis change |
| 22 | How do you document familial disease history? | Multiple choice |

# Clinical Impact Scale (1 = strongly disagree, 5 = strongly agree)

| **Item** | **Question** | **Response options** |
| --- | --- | --- |
| 23 | Family history meaningfully affects my risk assessment. | 1 / 2 / 3 / 4 / 5 |
| 24 | Family history affects my testing/screening decisions. | 1 / 2 / 3 / 4 / 5 |
| 25 | Family history affects treatment initiation/intensity. | 1 / 2 / 3 / 4 / 5 |
| 26 | Family history affects referral/genetic counseling decisions. | 1 / 2 / 3 / 4 / 5 |

# Recent clinical decisions and case scenarios

| **Item** | **Question** | **Response options** |
| --- | --- | --- |
| 27 | In the last 3 months, which decisions did you change because of family history? | Multiple choice |
| 28 | Approximately how many patients in the last 3 months were affected by family history in your decision-making? | 0 / 1-5 / 6-10 / 11-20 / >20 |
| 29 | Which decisions are influenced most by family history? | Multiple choice, up to 3 selections |
| 30 | Can you classify risk (high/medium/low) based on family history? | Yes, mostly / Sometimes / No |
| 31 | Which approach do you use to follow or refer high-risk patients? | Official guidelines / My clinical experience / Intuitive decisions / Clinical scenarios |
| 32 | Case scenario: 45-year-old man, LDL 165 mg/dL, BP 130/80, non-smoker, father with MI at age 49. What would you do? | Multiple choice |
| 33 | Case scenario: 38-year-old woman, asymptomatic, maternal aunt diagnosed with breast cancer at age 43. What would you do? | Multiple choice |
| 34 | Case scenario: 52-year-old man, asymptomatic, brother diagnosed with colorectal cancer at age 54. What would you do? | Multiple choice |

# Perceived Barriers Scale (1 = not at all, 5 = very much)

| **Item** | **Question** | **Response options** |
| --- | --- | --- |
| 35 | Time constraint | 1 / 2 / 3 / 4 / 5 |
| 36 | Reliability of patient-provided information | 1 / 2 / 3 / 4 / 5 |
| 37 | Lack of an appropriate documentation field/template | 1 / 2 / 3 / 4 / 5 |
| 38 | Access to up-to-date guideline information | 1 / 2 / 3 / 4 / 5 |
| 39 | Privacy/ethical concerns | 1 / 2 / 3 / 4 / 5 |
